# Effect of Silicon‐Based Electrolyte Additive on the Solid‐Electrolyte Interphase of Rechargeable Mg Batteries

**DOI:** 10.1002/advs.202510456

**Published:** 2025-10-27

**Authors:** Shivaraju Guddehalli Chandrappa, Guruprakash Karkera, Sirshendu Dinda, Mario Löw, Holger Euchner, Adam Reupert, Soutam Panja, Mohan K Bhattarai, Matthias M May, Zhirong Zhao‐Karger, Maximilian Fichtner

**Affiliations:** ^1^ Helmholtz Institute Ulm for Electrochemical Energy Storage (HIU) 89081 Ulm Germany; ^2^ Institute of Inorganic Chemistry II Ulm University 89081 Ulm Germany; ^3^ Institute of Theoretical Chemistry Universität Ulm D‐89081 Ulm Germany; ^4^ Institute of Physical and Theoretical Chemistry D‐72076 Tübingen Germany; ^5^ Department of Physics University of Puerto Rico San Juan PR 00931 USA; ^6^ Institute of Nanotechnology Karlsruhe Institute of Technology (KIT) 76021 Karlsruhe Germany

**Keywords:** electrolyte additive, rechargeable Mg batteries, solid‐electrolyte interface

## Abstract

The unstable solid‐electrolyte interface (SEI) poses a major obstacle to the widespread use of rechargeable magnesium batteries (RMBs) as high‐volumetric‐capacity next‐generation energy storage systems. This issue is effectively mitigated by adding 3 wt.% tris(trimethylsilyl) borate (TMSB, C9H27BO3Si3) to a state‐of‐the‐art Cl‐free magnesium tetrakis(hexafluoroisopropyloxy)borate in dimethoxyethane (Mg[B(hfip)_4_]_2_/DME) non‐aqueous electrolyte. The modified electrolyte enables stable Mg||Mo_6_S_8_ (Chevrel phase, CP) full cell operation for up to 1000 cycles at a 1C rate. Tip‐enhanced Raman spectroscopy (TERS) reveals that TMSB scavenges degraded electrolyte components and facilitates the formation of a uniform and thin SEI on the magnesium anode. Reflection anisotropy spectroscopy (RAS) further demonstrates that TMSB transforms the interfacial structure, creating a more isotropic and robust SEI during the initial stripping and plating process, thereby extending electrochemical cycling stability. This approach presents a compelling pathway for practical RMB development by stabilizing the SEI and optimizing magnesium electrolyte formulations.

## Graphical abstract

In this work, a silicon‐based electrolyte additive is introduced to condition the interfaces through an activation process. Symmetric cell studies with the additive show a five‐fold decrease in interfacial resistance and over 1200 h of stable Mg plating/stripping overpotential. The full cells with Mo_6_S_8_ cathodes exhibit excellent cycling stability over 1000 cycles at a 1C rate.

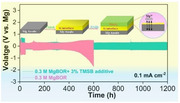

## 1 Introduction

Due to the high abundance of Mg (1.94% vs 0.002% for Li in the earth crust), the low electrode potential (−2.4 V vs SHE), and the high theoretical volumetric capacity (3833 mA h cm^−3^ for Mg vs 2062 mA h cm^−3^ for Li), RMBs are among the most promising post‐Li batteries.^[^ ^1^, ^2^, ^3^ ^]^ However, a significant issue with RMBs is the passivated interphase layer at the Mg metal side, which results from parasitic side reactions between the Mg metal anode and a majority of conventional electrolytes, and contaminants,^[^ ^1^ ^]^ leading to subpar lifespan and capacity.^[^ ^4^ ^]^ This is due to the fact that, unlike the SEI in common lithium‐ion batteries (LIBs) that conducts Li^+^, most SEIs generated on magnesium metal obstruct the transport of Mg^2+^.^[^ ^4^ ^]^

To alleviate this issue, it is essential to build an active SEI layer that promotes reversible Mg^2+^ ion conductivity to enhance the performance of current Mg batteries.^[^ ^3^, ^5^, ^6^ ^]^ Intrigued by these observations, many research groups have shown that it is possible to activate Mg anodes by adding inorganic halides (such as MgCl_2_),^[^ ^7^, ^8^, ^9^, ^10^, ^11^, ^12^ ^]^ which however, result in poor cycling stability due to the high corrosive behavior, low anodic stability, and low solubility of chloride‐based salts.^[^ ^13^, ^14^ ^]^

Recently, Chinnadurai et al.^[^ ^3^ ^]^ reported the use of MgBr_2_ as an electrolyte additive with magnesium bis(hexamethyldisilazide) (Mg(HMDS)_2_) in a 1,2‐dimethoxyethane (DME) electrolyte system. They found that the presence of the electrolyte additive modifies the Mg anode‐electrolyte interface, thus promoting the Mg‐ion bi‐directional transport and preventing anode passivation. Similarly, Li et al.^[^ ^15^ ^]^ investigated the effect of the SbCl_3_ electrolyte additive with 0.3 m Mg(OTf)_2_ and 0.2 m MgCl_2_ in DME on the electrochemical performance of Mg batteries. The authors ascribe better interfacial kinetics and electrochemical performance to the formation of an Sb‐based artificial interphase layer containing mostly MgCl_2_ and Mg_3_Sb_2_ on the Mg anode surface. In another case, Zhang et al.^[^ ^16^ ^]^ constructed an in vivo Ge‐based protective layer on the surface of Mg metal by incorporating GeCl_4_ into the Mg(TFSI)_2_/DME electrolyte. A route for quick Mg^2+^ transport is established by the Ge‐based protective layer, which also stops the passivation film from forming on the Mg anode surface. Yang et al.^[^ ^17^ ^]^ developed a simple method for creating magnesiophilic In/MgIn sites on an Mg metal surface using an InCl_3_ electrolyte additive with 0.3 m Mg(OTf)_2_ in the DME electrolyte system. The authors claimed that the existence of such magnesiophilic sites, can control the Mg deposition behavior with flat and uniform Mg deposition morphology, which was identified as being the cause of the quick electrochemical response kinetics. Recently, Shin et al.^[^ ^18^ ^]^ developed a Ga‐rich protective layer on Mg metal via a galvanic replacement reaction between Mg metal and a GaCl_3_ solution with 0.5 m Mg(TFSI)_2_ in diglyme and tetraglyme electrolyte. It led to the establishment of a durable Ga‐rich protective layer on Mg metal, protection from surface passivation, and resulted in reversible Mg plating/stripping with substantially decreased overpotential in ether‐based electrolytes. However, the exact chemical compositions, the structure of the involved interfaces and their evolution under operating conditions is often not (fully) understood, calling for novel state‐of‐the‐art interface‐sensitive characterization methods such as TERS and operando reflection anisotropy spectroscopy.^[^ ^19^, ^20^ ^]^

Recently, the Cl‐free Mg[B(hfip)_4_]_2_/DME (MgBOR/DME) electrolyte has been demonstrated to be suitable for Mg batteries due to its high oxidative stability (≈4 V vs Mg), high ionic conductivity, and excellent Coulombic efficiency (CE) in Mg deposition.^[^ ^21^, ^22^, ^23^ ^]^ These features of the MgBOR salt make it the most promising among the current state‐of‐the‐art chlorine‐free magnesium electrolytes.

However, during cycling, the Mg anode in the MgBOR/DME electrolyte it exhibits significantly increased plating/stripping overpotential and a large initial interfacial impedance, which is observed during the initial electrochemical cycles. Additionally, full‐cell studies with Mg||Mo_6_S_8_ show lower capacity during the initial cycles. To mitigate these issues, our group's previous studies have shown that using functional electrolyte additives lowers the initial interfacial impedance and maximizes the initial charge/discharge capacity by conditioning the interfaces via an activation process.^[^ ^24^, ^25^ ^]^

In this study, we developed a silicon‐based protective layer on the surface of magnesium metal by incorporating TMSB as an additive into a Cl‐free Mg[B(hfip)_4_]_2_/DME (MgBOR/DME) electrolyte. The inclusion of the Si‐based additive significantly reduces the interfacial resistance compared to cells without the additive. When a Si‐based, SEI‐protected Mg anode is paired with a Mo_6_S_8_ cathode in a full cell, it demonstrates extended and improved electrochemical performance in comparison to unprotected Mg metal. The underlying mechanisms of TMSB's beneficial effects were investigated through detailed surface chemistry analysis of the Mg electrode, using a combination of conventional impedance spectroscopy, X‐ray photoelectron spectroscopy (XPS), micro‐Raman spectroscopy, and advanced techniques such as TERS, and RAS.

## 2 Results and Discussion

### 2.1 Electrochemistry of Mg[B(hfip)_4_]_2_/ DME Electrolyte with TMSB Additive

**Figure 1:**
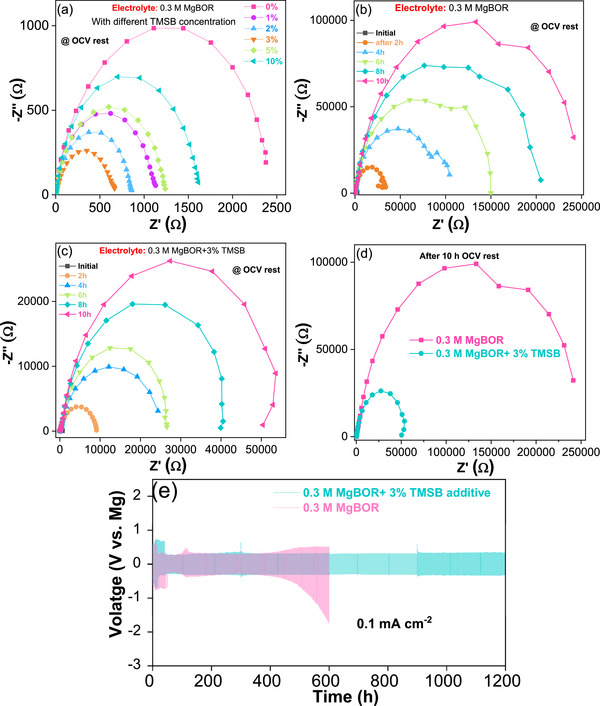
Mg/Mg symmetric cell studies in the 0.3 m MgBOR/DME electrolyte with and without additive: a) Impedance at OCV condition and for different additive concentrations, b) without additive at different rest hours c) with 3% TMSB additive at different rest hours, d) comparison of impedance spectra after 10 h rest at OCV with and without additive and e) Mg plating/stripping performance of the symmetric Mg/Mg cells in the 0.3 m MgBOR/DME electrolyte without additive and with 3% of TMSB at 0.1 mA cm^−2^ current density.

The effect of the additive on Mg plating/stripping was first investigated by symmetric cell studies in electrolytes that include the TMSB additive in 0.3 m MgBOR in DME solvent. **Figure** **1**a displays the Nyquist plots obtained under open‐circuit voltage (OCV) conditions of the symmetrical Mg/Mg cell with different concentrations of the TMSB additive.

An important factor influencing the interfacial resistance in the electrolyte is electrochemically inactive species that adsorb on the magnesium surface.^[^ ^24^ ^]^ As illustrated in Figure 1a–d, electrochemical impedance spectra (EIS) of a symmetrical Mg/Mg cell were acquired at various rest durations to monitor changes in the interfacial conditions. Using a pure MgBOR electrolyte at OCV, yielded a substantial interfacial resistance of ≈2.4 kΩ in a fresh cell. This resistance rose significantly with increasing resting time (Figure 1b), reaching 243 kΩ during a 10 h resting period. The interfacial impedance of the cell with the 3% TMSB also rose during a 10 h resting period (Figure 1a), finally amounting to 50 kΩ. This impedance is ≈5 times lower than for the case of the electrolyte without TMSB. The similar Nyquist plot behavior of the cell with TMSB corroborates that the additive significantly reduced the interfacial transport resistance, while forming a SEI with conductive components.

To further evaluate the effect of the TMSB additive on the stability of the Mg metal anode, Mg‐Mg symmetric cells were tested at a plating/stripping current density of 0.1 mA cm^−2^ for 1 h (Figure 1e; Figures S1 and S2, Supporting Information). The initial plating/stripping overpotential is high (≈ 0.7 V) for both cells, stabilizing after 40 h, at ≈ 260 mV. During the initial 10 h, the plating/stripping curves of the additive‐free cell are less smooth and show higher overpotentials compared to the cell with the additive, indicating unstable Mg deposition. These large overpotentials suggest sluggish charge transfer at the anode, likely due to the native oxide layer on the fresh Mg foil.^24^ However, after 400 h, the overpotential for the additive‐free cell increases significantly and reaches a much higher value of ≈ 2 V after 600 h. In contrast, the plating/stripping overpotential for the cell with an additive remains almost unchanged even after 1200 h. Again, the enhancement of the Mg plating/stripping behavior in the electrolyte with TMSB indicates that the additive promotes the formation of a favorable solid electrolyte interphase layer on the Mg surface.

### 2.2 Effect of TMSB on the Mo_6_S_8_‐Mg Full Cell Performance

**Figure 2:**
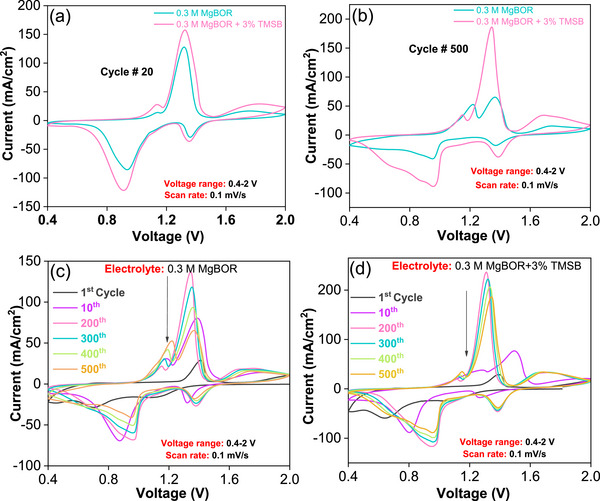
The Mo_6_S_8_‐Mg full cells electrochemical performance. a) CV comparison after the 20th cycle and b) after the 500th cycle for both electrolytes c) CV stability for the electrolyte without an additive, and d) CV stability for the electrolyte with additive at a scan rate of 0.1 mV s^−1^ and for a voltage window of 0.4–2 V.

As a next step, the effect of the TMSB additive on the electrochemical performance of the Mo_6_S_8_‐Mg full cell was investigated. The stability and compatibility of both electrolytes with the Mo_6_S_8_ cathode were examined through cyclic voltammetry (CV) (**Figure** **2**).

According literature,^[^ ^26^ ^]^ the insertion of Mg^2+^ in Mo_6_S_8_ involves two steps, corresponding to the formation of MgMo_6_S_8_ and Mg_2_Mo_6_S_8_, respectively. In both cases, after the 20th cycle, two distinct cathodic peaks were observed at 0.94 and 1.35 V versus Mg/Mg^2+^. Similarly, two anodic peaks at 1.32 V (Mg_2_Mo_6_S_8_ to MgMo_6_S_8_) and 1.78 V (MgMo_6_S_8_ to Mo_6_S_8_), corresponding to Mg de‐insertion, were observed. Notably, after 500 cycles, the cell with the additive exhibited minimal changes in peak intensity and position. In contrast, the additive‐free cell showed a noticeable shift and a decrease in both the anodic peak position and intensity (Figure 2b–d). This suggests the establishment of well‐stabilized charge transfer kinetics in the cell with TMSB.^[^ ^27^ ^]^

**Figure 3:**
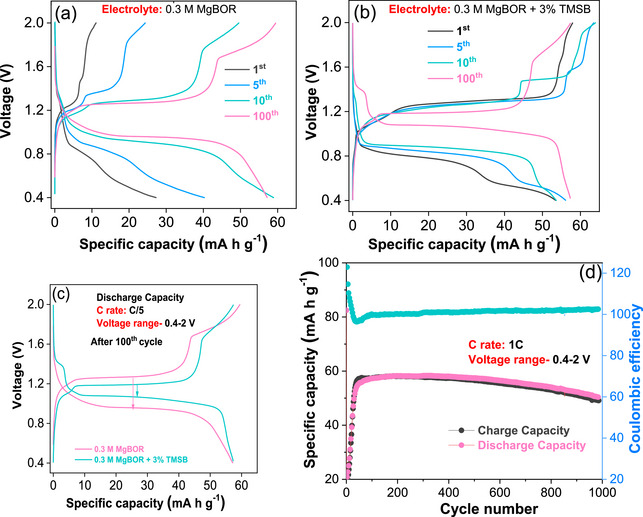
Electrochemical performance of the Mo_6_S_8_‐Mg full cell: a) discharge/charge profiles of the cell without additive, b) discharge/charge profile of the cell with additive c) discharge/charge voltage hysteresis comparison and d) cyclic stability of the cell with 3% TMSB at 1C rate.

**Figure** **3**a depicts the charge‐discharge profile of the additive‐free cell, conducted at a current rate of C/5 within a voltage range of 0.4–2 V. In the initial cycle, the cell demonstrates a discharge capacity of 25 mA h g^−1^ with a CE of 42%. By the end of the fifth subsequent cycle, it retains a capacity of 40 mA h g^−1^with a CE of 60%. Subsequently, the capacity stabilizes after the tenth cycle, reaching 59 mA h g^−1^ with a CE of 84%, which is retained, also after the 100th cycle.

With additive, the cell shows a higher discharge capacity of 54 mA h g^−1^ with a CE of 109% during the initial cycle and 57 mA h g^−1^ for the 5th cycle. After the 100th cycle, it retains a discharge capacity of 56 mA h g^−1^ with a high CE of 100% (Figure 3b). These results confirm that the bare electrolyte requires a few initial cycles for conditioning of the interfaces. This is likely to be due to the slow occupation of the inner‐ring site of Mo_6_S_8_ during the activation cycles. Once the inner‐ring sites are occupied, the activation process concludes. From that point onward, both the capacity and Coulombic efficiency stabilize.^[^ ^24^ ^]^ The cell with TMSB reaches faster its full capacity. Interestingly, additive‐free cells show a voltage hysteresis of 0.3 V after 100 cycles. In contrast, the cell with TMSB demonstrates a significantly lower hysteresis of 0.1 V, highlighting the notable impact of the additive (Figure 3c) in effectively alleviating voltage hysteresis and improving electrochemical performance. Similar reductions in the charge–discharge voltage hysteresis were observed for the use of H_3_PO_4_/SiCl_4_ additives in Mg metal batteries.^[^ ^28^ ^]^ Furthermore, the long‐term cycle stability of the Mo_6_S_8_ cathode/electrolyte/Mg anode cell with TMSB was evaluated at a higher rate of 1C (Figure 3d). In the initial cycle, the cell exhibited a discharge capacity of 23 mA h g^−1^. After 20 cycles, this capacity increased to ≈40 mA h g^−1^ with 100% CE. Remarkably, after ≈1000 cycles, the discharge capacity reached 50 mA h g^−1^ with a CE close to 100%, showcasing superior cycling stability. Here, it has to be pointed out that the cycling stability of the Mo_6_S_8_ cathode with the TMSB additive outperforms several reported additive‐based studies, hence highlighting the remarkable role of the proposed additive in Mg batteries (Table S2, Supporting Information). Similarly, a comparison of the cycling stability of Mo_6_S_8_ cathode/electrolyte/Mg anode cells with and without the TMSB additive was evaluated at a current rate of C/5, as shown in Figure S3 (Supporting Information).

### 2.3 Raman Studies

To critically evaluate the effectiveness of TMSB as an additive and to gain a deeper understanding of SEI formation, we conducted micro‐Raman and TERS studies on the Mg working electrode (WE) in a Mg–Mg symmetric cell. Details of the sample preparation and Raman experimental procedures are provided in the experimental section. Briefly, both micro‐Raman and TERS techniques probe the molecular fingerprints of surface species. However, while micro‐Raman provides an average measurement over a volume of a few µm^3^ and therefore often fails to obtain sufficient Raman intensity from a thin SEI layer, TERS, being an enhanced Raman technique, captures nanoscale chemical information from a volume of only a few nm^3^. This enhancement overcomes the low Raman scattering cross‐section of thin SEI layers.

**Figure 4:**
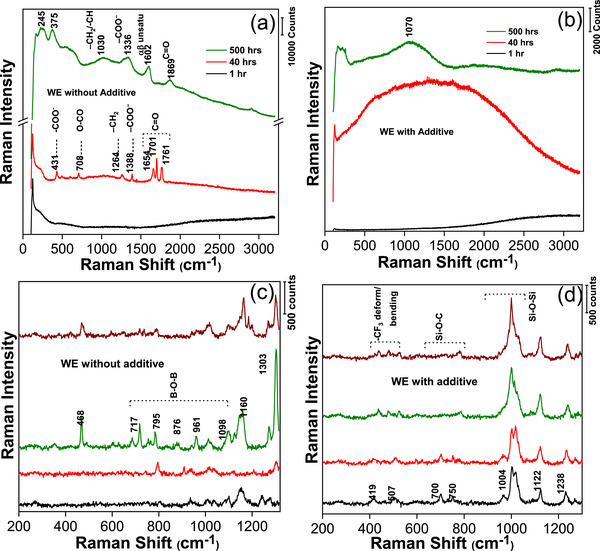
a,b) Micro‐Raman spectra of the Mg working electrode surface. a) Surface without the additive, showing continuous evolution of the surface layer with subsequent cycling. b) Surface with the TMSB additive, indicating significantly slower SEI progression and a notably lower presence of SEI debris. c,d) TERS spectra from various locations on the Mg working electrode surface, after 40h cycling from a symmetric cell. c) Without the additive, the spectra reveal a significant presence of B‐O‐B segments associated with magnesium borate compounds. d) With the additive, the spectra indicate the formation of distinct organic siloxanes and silanols, which are not detected in the micro‐Raman spectra.

Initially, we used micro‐Raman to study the Mg WE surface of the symmetric cell after the 1st, 40th, and 500th h (see **Figure** **4**a,b). The results reveal notable differences between the additive‐free and additive‐containing cells. The WE surface without TMSB (Figure 4a) shows significant changes throughout the cycling process, whereas the WE surface with the TMSB additive (Figure 4b) exhibits minimal spectral signatures after the 40th h. This indicates that the surface layer formed in the absence of TMSB is significantly thicker, as demonstrated by the stronger and more pronounced Raman intensities. The SEI components primarily consist of oxalate and acetate salts of Mg, evolving with continued cycling into anhydrides and αβ‐unsaturated carboxylates. A detailed analysis of the Raman spectra is provided in the section Micro‐Raman Study of the supporting information. As a significant shift in the stripping and plating behavior was observed after the 40th cycle, we conducted TERS measurement, at different local points, on the reacted Mg surfaces, retrieved from the cells with and without TMSB. TERS provides highly localized chemical recognition, specifically focused on the thin surface layer, offering a clearer qualitative understanding of the SEI formation and its composition. Figure 4c,d illustrates the TERS spectra collected from four distinct points on each electrode surface. These spectra, recorded in the low‐frequency range of 200–1310 cm^−1^ (see Figure 4a,b), reveal the formation of borate‐ and silicon‐based inorganic and organic compounds, offering valuable insights into the degradation chemistry and the impact of the TMSB additive.

**Scheme 1:**
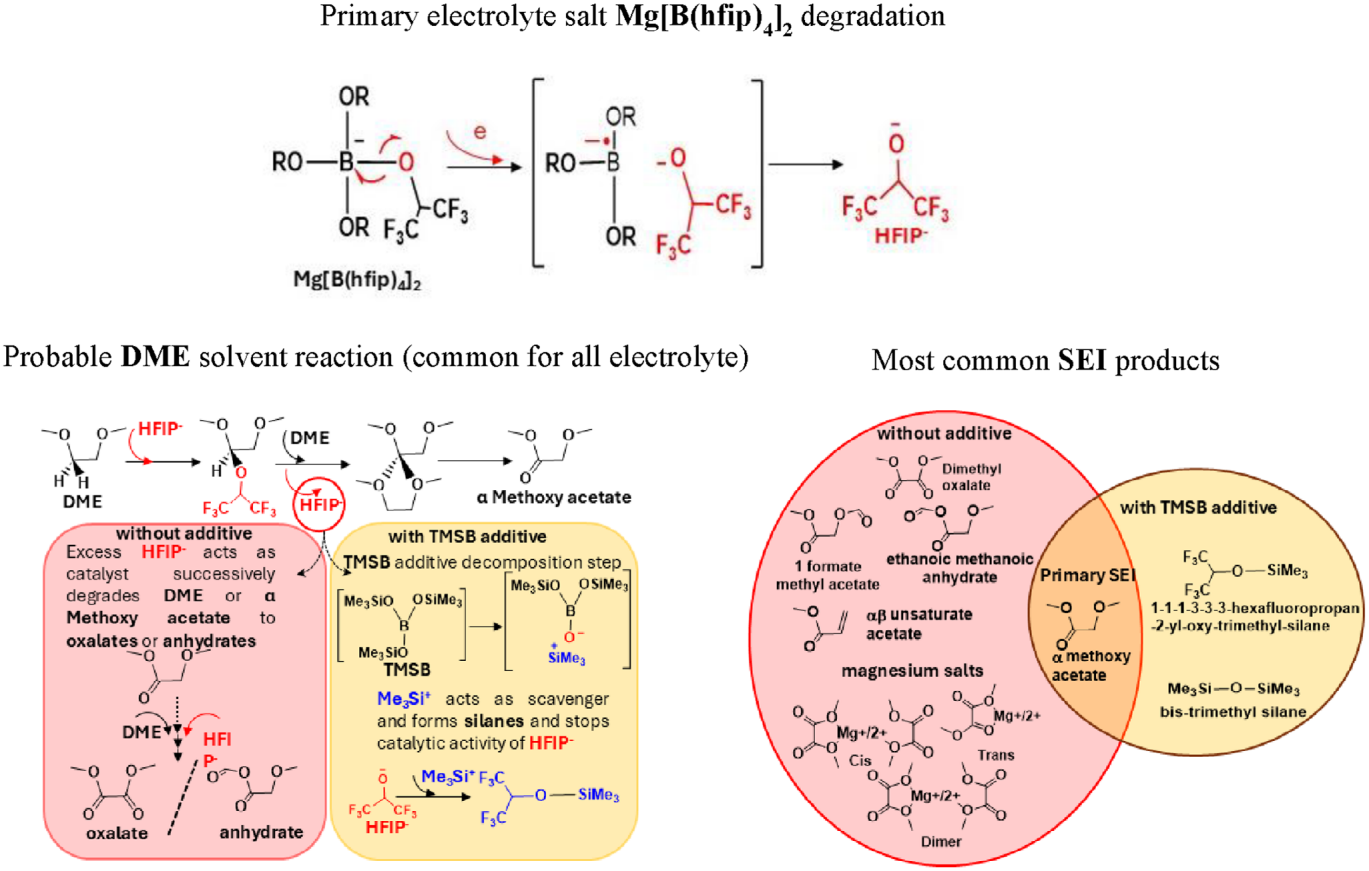
Revealing multiple potential degradation mechanisms and the presence of various organic and inorganic salts on the SEI layer for surfaces with and without the additive.

For the WE without TMSB, the TERS spectra display prominent and sharp Raman bands at 1303, 1160, 795, 717, and 448 cm^−1^. These peaks are characteristic of SEI fragments. Specifically, the pronounced bands at 1160, 795, and 717 cm^−1^ as well as those at 1098 and 961 cm^−1^ can be attributed to various vibrational modes of B─O bonds in magnesium borate compounds,^[^ ^29^, ^30^ ^]^ plausibly Mg_3_(BO_3_)_2_. On the other hand, the TERS spectra from the Mg electrode with TMSB show vibrational bands at 1238, 1122, 1004, 750, 700, 507, and 419 cm^−1^. Among these, the most intense peak near ≈1000 cm^−1^ is likely to correspond to the Si‐O‐Si asymmetric stretching, while the band at ≈419 cm^−1^ may represent rocking modes of siloxanes.^[^ ^31^, ^32^ ^]^ The Raman bands at 750 and 700 cm^−1^ are assigned to C‐Si‐O and Si‐O‐C bending modes of aliphatic silanes/silanols. Additional features include the bands at 1238 and 1122 cm^−1^, which may correspond to C‐CF_3_ stretching vibrations, and the band at ≈507 cm^−1^, attributed to CF_3_ vibrations^[^ ^33^, ^34^, ^35^, ^36^ ^]^ (see **Scheme** **1**). In summary, the SEI composition significantly differs in the presence of TMSB, consisting primarily of siloxanes and silanols. This analysis underscores the critical compositional differences in the SEI layers, shedding light on the role of TMSB in influencing compositional/surface chemistry and stability.

The TERS observations suggest that Mg[B(hfip)_4_]_2_ initially decomposes to release hexafluoroisopropoxide (HFIP^−^) anions and magnesium borate inorganic salts, as evidenced by the B‐O vibrational modes^[^ ^21^ ^]^ (see Figure 4c and Scheme 1, Electrolyte Decomposition). We propose that HFIP^−^ acts as a catalyst, first degrading the solvent dimethoxyethane (DME) into α‐methoxy acetates (see Scheme 1, Probable reaction). In the absence of TMSB, HFIP^−^ promotes multi‐step degradation, further converting DME into oxalates, anhydrates, and αβ carboxylates, as confirmed by the micro‐Raman results after 500 cycles (Figure 4a).^[^ ^37^ ^]^ When TMSB is introduced, it undergoes degradation to form trimethyl silicon (Me_3_Si^+^). These cations scavenge HFIP^−^, halting its catalytic activity and preventing further degradation of DME. Furthermore, residual TMSB degradation in subsequent cycles leads to the formation of siloxane‐based compounds, resulting in the broader Raman band observed at ≈1070 cm^−1^ (Figure 4b). The proposed mechanism underscores the crucial role of TMSB in minimizing solvent degradation and designing a stable, conductive SEI to mitigate the Mg anode passivation, as reflected by enhanced symmetric cell tests and durability of the Mo_6_S_8_‐Mg cells.

### 2.4 Reflection Anisotropy Spectroscopy

Since the TERS data show the formation of an interphase on top of the Mg anode, RAS was applied for the in operando investigation of the initial steps of the SEI formation process for electrolytes with and without an additive during electrochemical cycling.

For this investigation, an Mg(0001) single‐crystal anode was used. Since Mg(0001) is optically isotropic, it should not provide an anisotropy signal. However, the crystal delivers a distinct RA signal, which we interpret in terms of a surface oxide. To test this hypothesis, we have investigated an MgO(110) single crystal via experimental and computational RAS, which indicates that the observed anisotropy signal indeed originates from a surface oxide on the Mg single crystal (see Figure S8, Supporting Information).

**Figure 5:**
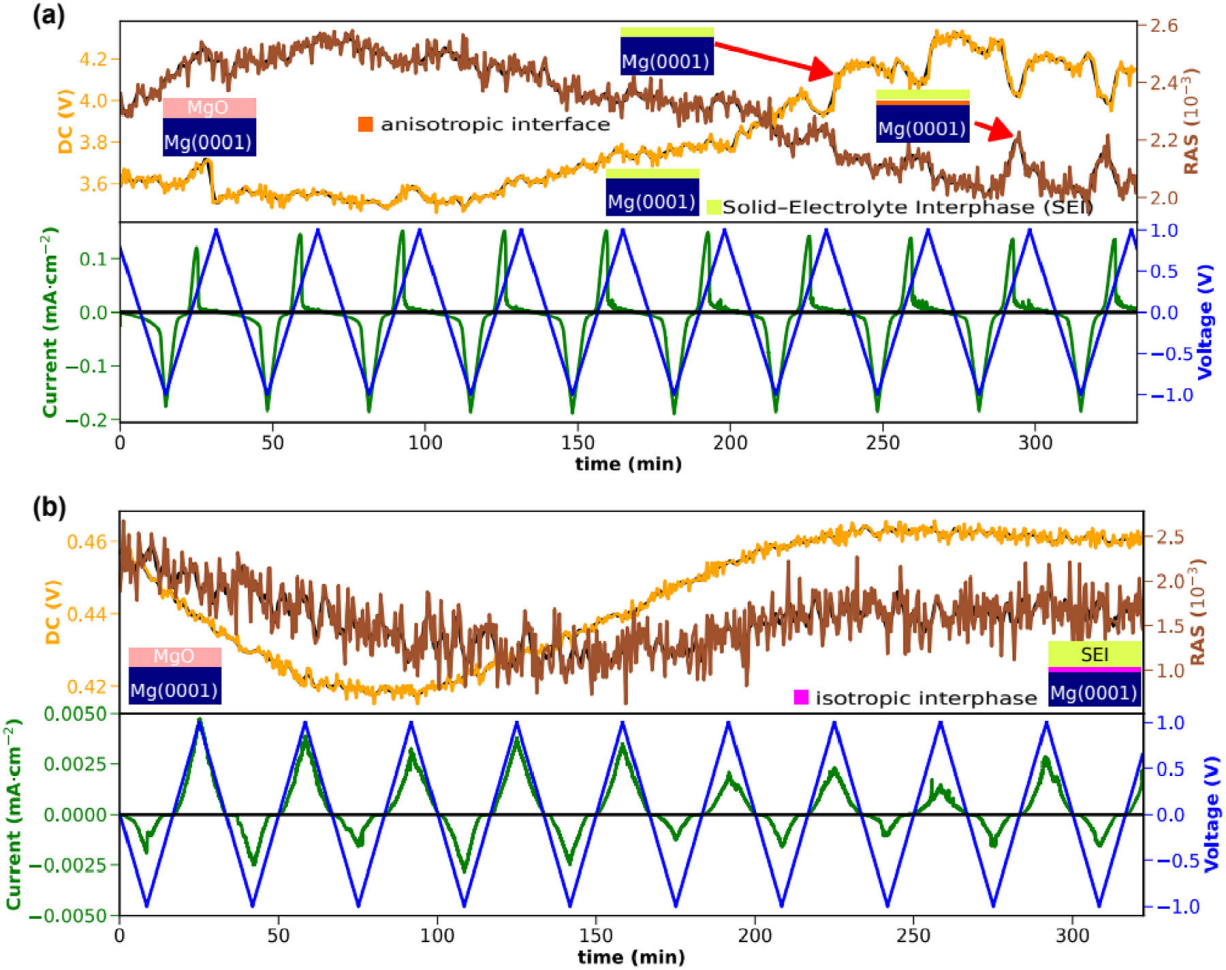
a) Changes of the anisotropy and reflectance during cycling without additive: The upper part shows a transient at 2.65 eV of the real part of the reflection anisotropy in brown and the DC signal (proportional to the reflectance, orange). The graphs were smoothed with a Savitzky–Golay–filter. The lower part shows the current density (green) and the corresponding applied voltage (blue). CV: ‐1–1 V (vs. Mg pseudo‐reference) with a scan rate of 2 mV s^−1^ for 10 cycles. b) Changes in the anisotropy and reflectance during cycling with additive. The upper part shows a transient at 2.65 eV, and the lower part shows the current density and the corresponding applied voltage of the CV. The insets represent sketches of the interface structure of the Mg‐metal anode while cycling.

**Figure** **5**a shows the time‐dependent change of the anisotropy and the corresponding reflectance during cycling in the MgBOR electrolyte without TMSB. The anisotropy (brown curve) increases in the first 30 min and stays constant; after ≈60–100 min, it declines slowly. At the same time, the DC signal (proportional to the reflectance) of the sample starts to increase. The decreasing RA signal and the increasing DC signal point to the (partial) dissolution of the native surface oxide on the magnesium crystal, since only MgO is expected to show an optical anisotropy. The periodic oscillations that start to appear after ≈200 min are more clearly visible in the DC signal, but are present in the RA response as well. Dips in the reflectance and local maxima in the anisotropy arise between the maximum of the anodic current and the maximum of the applied anodic potential. The local minima or dips in the reflectance indicate that in the potential window between 0.5 and 1.0 V versus Mg, either a (micro)roughening of the electrode surface occurs, or an interfacial or interphase layer develops which changes the reflectance at the given photon energy. The simultaneous rise of a local optical anisotropy maximum, on the other hand, means that some degree of ordering is involved here. This could therefore be an anisotropic (ordered) stripping or the (re)formation of an interfacial layer with reduced reflectance, but a minimum degree of ordering, such as oxide re‐formation.^[^ ^38^ ^]^ Consequently, these quasi‐periodic oscillations are an indicator of an almost reversible process that probably originates from unhindered and anisotropic metallic stripping and plating and/or the potential‐dependent formation‐dissolution of an ordered interfacial (oxide) layer. This means that after an initial conditioning period, an intermediate, anisotropic interfacial layer evolves that forms a relatively sharp interface to the bulk and changes during cycling.

As depicted in the lower part of the figure, the current density shows periodic stripping and plating for the whole time span. The corresponding CVs (see Figure S7, Supporting Information) do not show significant changes after the first cycle. These observations corroborate the picture of MgO dissolution followed by metallic stripping/plating and SEI formation.

In the case of the additive‐containing electrolyte, the behavior of the Mg single crystal is completely different, and no quasi‐reversible change in RA or DC signal is observed (Figure 5b) during the initial cycles. Here, the optical anisotropy does not show significant changes, yet the reflectance undergoes a minimum during the first three cycles, recovering to values slightly above the initial reflectance after seven cycles. This observation is similar to the overall long‐term trend of the sample without an additive, supporting the hypothesis that during these cycles, an SEI with higher reflectance is formed. While this makes an interpretation of the processes at the solid‐liquid interface difficult, the fact that the additive‐free electrolyte shows a completely different behavior clearly points to the significant impact of the additive on the SEI growth, promoting a more isotropic structure at the interface between SEI and bulk Mg.

One has to keep in mind that significant changes in the plating/stripping behavior of the cell are only observed beyond 40 h (see Figure 1e). The measurement with RAS, however, only shows the first 5 h during CV. Consequently, this period is too short to monitor all processes occurring during long‐term cycling and is limited by the cell design that requires considerable amounts of the electrolyte. Furthermore, the RAS measurements must use Mg single crystals, whereas (polycrystalline) magnesium foil was used in the other experiments. Yet the qualitative difference between the operando RAS observations for electrolytes with and without TMSB is evident and suggests that the stripping and plating in this initial time frame form more optically anisotropic –, i.e., ordered – interfaces without additive. In contrast, the interface between SEI and Mg is more isotropic with TMSB. This indicates that the additive promotes structural changes during plating and stripping that are spatially more extended toward the electrode bulk, while in the case without TMSB, sharper phase boundaries are maintained. This can be seen in the oscillation of the reflectance and the optical anisotropy, which stems from the stripping and plating process. In the case of the electrolyte with an additive, these periodic oscillations do not occur, but the change in reflectance over several hours suggests the slow formation of an SEI. The difference in the current density for the experiments on the single crystals most probably arises from a denser SEI on these very planar surfaces with an associated lower ion conductivity.

### 2.5 Ex Situ Analysis of the Cathode

To better understand the charge transfer mechanism at the Mo_6_S_8_ cathode, we have performed ex situ XPS analysis of the electrodes after the first discharge and charge. Figure S5a,b (Supporting Information) shows the Mo 3d spectra of the discharged/charged cathode, which can be deconvoluted into two doublets. The lower binding energy doublets are assigned to Mo^3+^ and Mo^2+^ oxidation states in the sulfur environment.^[^ ^39^ ^]^ The corresponding binding energy values are listed in Table S1 (Supporting Information).

Upon magnesiation, (Figure S5a, Supporting Information), there is no evolution in the Mo 3d_5/2‐3/2_ core peak signature in both additive and additive‐free electrolytes. However, in the case of the additive‐containing cell, the intensity of the Mo^3+^ peak decreases while the intensity of the Mo^2+^ peak increases. This suggests that the additive could enhance the Mg intercalation process. During demagnesiation, (Figure S5b, Supporting Information), the peak of Mo^3+^ doublets increases and Mo^2+^ decreases, suggesting that Mo^2+^ is oxidized back to Mo^3+^ in the first charge.^[^ ^40^ ^]^

Interestingly, the cell with additive shows a higher intensity of Mo^3+^ doublets than without the additive, indicating that the additive further improves the Mg de‐intercalation process. Similarly, the S 2p peaks can be fitted with two doublets (Figure S5c,d, Supporting Information): one representing 4‐ coordinated S^2−^ attributed to sulfur coordinated by four Mo atoms (three from the same Mo_6_ cluster and one from a neighbouring cluster, labeled as peripheral sulfurs), and the other representing 3‐coordinated S^2−^ attributed to sulfur coordinated by three Mo atoms (same Mo_6_ cluster, labeled as axial sulfurs).^[^ ^39^, ^40^ ^]^ During the discharge process, the 3‐coordinated S^2−^ doublet completely disappears, whereas 4‐coordinated S^2−^ doublets emerge, which may be a consequence of the increasing distance between the clusters due to Mg insertion. Similarly, during the charge process, 3‐coordinated S^2−^ doublets reemerge in both electrolyte systems. However, the additive cell shows a higher intensity of both 3‐ and 4‐coordinated S^2−^ doublets, indicating that the Mg inter/de‐intercalation process in the presence of the additive is highly reversible as compared to the additive‐free electrolyte. Similarly, the results for the ex situ analysis of the anode are presented in Figure S6 (Supporting Information), and the corresponding discussion is provided in the Supporting Information.

## 3 Conclusion

In summary, we demonstrated TMSB as an effective additive to design a novel and functional solid‐electrolyte interphase for Mg metal batteries. TMSB facilitates reversible Mg intercalation into theMo_6_S_8_ for a typical Cl‐free Mg[B(hfip)_4_]_2_/DME (MgBOR/DME) based non‐aqueous electrolyte. On the other hand, TMSB scavenges degraded electrolyte components and facilitates the formation of a uniform and thin SEI on the magnesium anode. This improvement allows the electrolyte to endure long‐term electrochemical cycling stably. With a 3% TMSB additive in the electrolyte, we observed a five‐fold decrease in interfacial resistance at 10 h OCV rest and over 1200 h of stable Mg plating/stripping overpotential in symmetric cell studies. Furthermore, the cell combining a Mo_6_S_8_ cathode with a 3% TMSB additive electrolyte exhibits stable Mg insertion/deinsertion behavior for 500‐cycles, as demonstrated by CV studies. Moreover, the cell shows excellent charge‐discharge cycling stability for up to 1000 cycles with low voltage hysteresis of only 0.1 V for high C rates. The current study presents a new promising avenue for constructing a stable SEI layer on the Mg anode by utilizing a functional electrolyte additive. This approach holds significant potential for enabling the practical use of high‐performance rechargeable magnesium batteries.

## 4 Experimental Section

### Electrolyte Preparation

Anhydrous 1, 2‐dimethoxyethane (DME, 99.5%, inhibitor‐free) was obtained from Sigma–Aldrich and dried using molecular sieves (3 Å, Fisher Chemical).

### Preparation of 0.3 m MgBOR/DME

The synthesis of the electrolyte salt Mg[B(hfip)_4_]_2_·3DME followed established methods.^[^ ^41^, ^42^ ^]^ Subsequently, a 0.3 m electrolyte solution was prepared by dissolving Mg[B(hfip)_4_]_2_·3DME in an appropriate volume of DME in a volumetric flask.

### Preparation of the Electrolyte with an Additive

The TMSB additive was added to the 0.3 m MgBOR/DME electrolyte to obtain the electrolytes with the additive concentrations of 1%, 2%, 3%, 5%, and 10%, respectively.

### Spectro‐Electrochemical Characterization

For structural operando characterization with electrochemical RAS, a Laytec EpiRAS was used in combination with a TSC Raman cell from RHD Instruments. All experiments were carried out in an Argon‐filled glovebox from MBRAUN (H_2_O and O_2_ < 1 ppm). For the operando electrochemical measurements, a Gamry Interface 5000 Potentiostat from Gamry was used.

RAS is a spectroscopic technique that allows for non‐destructive examination of the electrode‐electrolyte interface, by analyzing the difference in reflectivity between two orthogonal directions in the surface plane (x, y).^[^ ^19^, ^43^ ^]^ To gain insight in the mechanism at the solid electrolyte interface, a single‐crystalline Mg (0001) surface was investigated in an electrochemical environment. As this crystal in principle does not show optical anisotropy, the detected RAS signal is a direct probe of the processes at the surface (and structural anisotropies in the topmost layers). For the electrochemical measurements, CV between −1 and 1 V (vs Mg pseudo‐reference) with a scan rate of 2 mV s^−1^ were recorded for ten cycles, starting from open circuit potential (OCP) toward the negative terminus. During the CV, multiple RA spectra for photon energies from 1.45 to 4.0 eV were recorded in series, yielding so‐called color plots. A zero‐line correction was applied to all spectra using an optical isotropic Si (100) wafer inside the TSC cell.

### Micro‐Raman and Tip‐Enhanced Raman Measurements

Micro‐Raman spectra of the cycled magnesium electrodes were recorded in the spectral range of 200–3200 cm^−1^ using an inVia confocal Raman microscope (Renishaw) equipped with a 633 nm excitation laser. The confocal system utilized a grating with a groove density of 1800 lines per millimeter, providing a spectral resolution of 1 cm^−1^. A 50X objective lens (0.75 numerical aperture, NA) in backscattering geometry was employed to collect the Raman spectra.

The TERS instrument is a state‐of‐the‐art commercial setup combining a Renishaw inVia Raman microscope with a Bruker Innova‐IRIS scanning probe microscope (SPM) via an extended external optical arm. The TERS system employs side illumination geometry, using a long working distance objective (50X, 0.42 NA) to focus the laser on the apex of the scanning tunneling microscope (STM) tip. Although the low NA objective and angular collection geometry reduce excitation and collection efficiency, this configuration is necessary for analyzing opaque or relatively thick surfaces. The SPM was operated in a glovebox atmosphere, allowing measurements on air‐ and moisture‐sensitive electrode‐electrolyte interface (EEI) surfaces.

To establish optical alignment and calibration, STM‐based TERS measurements were performed on a commercial malachite green sample deposited on a gold substrate. A gold‐etched IRIS STM tip from Bruker was used, with a 633 nm laser providing an electromagnetic excitation source that achieved an enhancement factor of ≈10^6^.^[^ ^20^ ^]^ The tunneling current was set at 1 nA, and the tip bias potential was maintained at 0.4 V. The tip was carefully approached to the sample surface, and the laser focal point was adjusted to optimize the “hot spot” at the tip apex. During measurements, the laser's input average power was 0.5 mW, with an exposure time of 1 s. A grating with a groove density of 1600 lines per millimeter was used for spectral dispersion.

Throughout the TERS measurements, the tunneling current (1 nA) and tip bias (0.4 V) were kept constant. Data collection was managed through the IRIS software, which integrates control of the Renishaw inVia Raman microscope with the Bruker Innova SPM system. Post‐processing, including background subtraction and cosmic ray removal was performed using a custom‐built MATLAB script.

## Conflict of Interest

The authors declare no conflict of interest.

## Supporting information



Supporting Information

## Data Availability

The data that support the findings of this study are available from the corresponding author upon reasonable request.
