# Supplementary material for: Effect of Silicon‐Based Electrolyte Additive on the Solid‐Electrolyte Interphase of Rechargeable Mg Batteries
Source: Adv Sci (Weinh). 2025 Oct 27;13(2):e10456. doi: 10.1002/advs.202510456 (PMC12786364; doi:10.1002/advs.202510456)
Supplement: Supplementary file 1 — Supporting Information [file ADVS-13-e10456-s001.docx]

Supporting Information

**Effect of silicon-based electrolyte additive on the solid-electrolyte interphase of rechargeable Mg batteries**

*Shivaraju Guddehalli Chandrappa,*^a^ Guruprakash Karkera,*^a^ Sirshendu Dinda ^a^,Mario Löw,^d^ Holger Euchner,^c^ Adam Reupert,^a^ Soutam Panja,^a^ Mohan K Bhattarai,^e^ Matthias M May,^c,d^ Zhirong Zhao-Karger,^a,b^ and Maximilian Fichtner *^a,b^*

1. **Electrode Preparation.**

**Mo_6_S_8_ Electrode Preparation.**

Mo_6_S_8_ powder (NEI Corporation), conductive carbon C65, and PVDF (Alfa Aesar) binder were mixed with a mass ratio of 7:2:1 in NMP to form a slurry. The slurry was cast onto stainless steel and dried. The diameter of the electrode is 11.8 mm. The loading of Mo_6_S_8_ on the electrode is 1.0-1.8 mg cm^-2^.

**Electrochemical Characterization.**

The coin cells were assembled inside an argon-filled glove box with polished Mg metal as the anode and a glass microfiber (Whatman GF/D) separator. Galvanostatic discharge/charge cycling was performed using a BioLogic VMP3 potentiostat. The CV measurements of Mg Vs. Mo_6_S_8_ with additive and pure electrolytes were examined from 0.2 to 2 V vs. Mg at a scanning rate of 0.1 mV s^-1^. Electrochemical impedance spectra (EIS) were measured from 1 MHz to 10 mHz. Galvanostatic Mg plating/stripping tests of Mg symmetric cells were conducted at a current density of 0.1 mA cm^-2^ for 0.5 h at each step.

**Characterization.**

X-ray photoelectron spectroscopy (XPS) measurements were carried out on a Specs XPS system with a Phoibos 150 energy analyzer using monochromatic Al Kα radiation (1486.6 eV), a take-off angle of 45°, and pass energies of 30 and 90 eV at the analyzer for detail and survey spectra, respectively. The samples were transferred under Ar from the glovebox to the XPS system to avoid contamination. CasaXPS was used for data analysis, using Shirley-type backgrounds and Gaussian- Lorentzian peak shapes. All spectra were calibrated to the C (1s) peak of adventitious carbon at 284.8 eV.

1. **Symmetric cell studies.**

**Figure S1**. Mg plating/stripping performance of the symmetric Mg/Mg cells in the 0.3 M MgBOR/DME electrolyte with 3% of TMSB and without additive electrolyte at 0.1 mA cm^-2^ current density.

**Figure S2**. Initial 10 hours of Mg plating/stripping performance of the symmetric Mg/Mg cells in the 0.3 M MgBOR/DME electrolyte with 3% of TMSB and without additive electrolyte at 0.1 mA cm^-2^ current density.

1. **Electrochemical Performance.**

**Figure S3.** Electrochemical performance of the Mo_6_S_8_-Mg full cell: cyclic stability comparison with 3% TMSB and without additive electrolyte cells at C/5 rate.

1. **Micro-Raman Study**

A strong solvation sheath can hinder Mg stripping and plating due to reduced ion mobility. To investigate this, we examined the solvation environment of 0.3 M MgBOR in DME, both with and without 3% TMSB additive. The normalized Raman spectra, shown in Figure D (750-940 cm^-1^ range), provide insight into these interactions. Pure DME exhibits distinct peaks at ~ 822 and 848 cm^-1^, corresponding to C-O stretching and CH_3_ wagging modes, respectively. The MgBOR salt alone shows a band at 801 cm^-1^ attributed to B-O stretching from the [B(hfip)_4_]^-^ anion, and a peak at 877 cm^-1^ linked to the symmetric breathing mode of DME molecules coordinating with Mg^2+^. Notably, the addition of TMSB does not shift the peak positions of these vibrational modes, indicating that TMSB does not significantly alter the solvation sheath energetics around either the cation or anion. However, an increase in the relative intensity ratio of the coordinated (877 cm^-1^) to free DME bands suggests enhanced DME coordination-implying a higher degree of Mg²⁺ solvation in the presence of TMSB^[1-3]^.

**Figure S4.** The 780-940 cm⁻¹ range, which corresponds to C-O stretching vibrations and -CH₃ wagging motions, as well as the presence of 'caged' or cation-associated DME.

Micro-Raman spectra from the Mg electrode without additive after the 40th cycle reveal prominent Raman bands at 1761, 1701, 1665, 1388, 1264, 708, and 431 cm⁻¹ **(see Figure 4(a)).** The bands at 1761, 1701, and 1665 cm⁻¹ correspond to the symmetric and asymmetric vibrations of the carbonyl (C=O) group, characteristic of carboxylates, αβ-unsaturated acetates/carboxylates, oxalates, and similar compounds. Notably, the absence of a strong Raman band in the 1580–1600 cm⁻¹ region (symmetric C=C stretching) rules out the presence of unsaturated acetates/carboxylates^[4-7]^. The Raman band at 1388 cm⁻¹ can be attributed to the symmetric stretching or asymmetric bending mode of the –COO⁻ group, while the 1264 cm⁻¹ band is associated with the wagging vibration of the –CH₂ group. The band at 708 cm⁻¹ corresponds to the bending vibration of O–CO or deformation of the –COO⁻ group. Finally, the 431 cm⁻¹ band signifies the rocking vibration of the –COO⁻ group or deformation of O–CO. Collectively, this Raman fingerprint suggests the formation of products such as dimethyl oxalate, methyl acetate, ethylene glycol diformate, and their ionic salts with magnesium **( Schematics 1).**

After 500 cycles, the micro-Raman spectra display broad bands at 1869, 1602, 1336, 1030, 375, and 245 cm⁻¹ **(see Figure 4(a)).** The high-frequency band at 1869 cm⁻¹ corresponds to the symmetric stretching of the carbonyl group in anhydrides, while the 1602 cm⁻¹ band is associated with the αβ-unsaturated segment or the asymmetric stretching of –COO⁻. The band at 1336 cm⁻¹ corresponds to the symmetric stretching of the –COO⁻ group, whereas the 1030 cm⁻¹ band is attributed to the asymmetric bending of –CH₃ or –CH₂ groups^[6-7]^. The bands observed around 245 and 375 cm⁻¹ are strong candidates for MgO-related vibrational features, particularly given their emergence only after prolonged cycling. These signals likely originate from defect-induced lattice vibrations in nanocrystalline or surface-modified MgO. Although bulk MgO is Raman inactive due to its centrosymmetric structure, such low-frequency bands can become active in the presence of phonon confinement effects or oxygen vacancies-phenomena commonly encountered in electrochemical and organometallic environments^[3, 6, 8]^. The progressive increase in intensity of bands within the 200-500 cm⁻¹ range (from 1 hr to 500 hrs) indicates the accumulation of surface modifications, structural defects, or the formation of MgO nanoparticles. Given that micro-Raman probes into sub-surface regions, these bands reflect both surface and near-surface chemical evolution. Notably, the Raman spectra of electrodes with the additive do not exhibit similar MgO-associated features, suggesting the absence of nanocrystalline MgO formation. This implies that the additive-TMSB-effectively suppresses or stabilizes the growth of MgO into larger nanostructure. These findings suggest that the solid electrolyte interphase (SEI) layer continues to evolve, with organic formates and oxalates progressively transforming into anhydrides, αβ-unsaturated carboxylates and magnesium salts of carboxylates/formates and considerable amount of MgO species^[6]^.

In contrast, the micro-Raman spectra of the working electrode (WE) with the TMSB additive after the 1st and 40th cycle do not display any significant Raman bands **(see Figure 4(b)).** This indicates that the SEI layer in the presence of the additive is much thinner compared to the electrode without the additive. As previously noted, micro-Raman spectroscopy is not sensitive to very thin layers. However, after 500 cycles, the spectra reveal a broad Raman band centered at approximately 1070 cm⁻¹, with no evidence of specific organic groups seen in the SEI without the additive.

1. **XPS- ex-situ analysis of the cathode**

| **Electrode** | **Mo^2+^** | **Mo^3+^** | **3-coordinated S** | **4-coordinated S** |
| --- | --- | --- | --- | --- |
| **Discharged** | With additive:  **B.E:** 227.24 and 230.41 eV  **R.I:** 47.27 and 3.05 % | **B.E:** 228.34 and 231.5 eV  **R.I:**43.07 and 4.13 % | **-** | **B.E:** 160.68 and 161.71 eV  **R.I:**21.51 and 78.49 % |
|  | Without additive:  **B.E:** 227.25 and 230.46 eV  **R.I:** 19.20 and 13.10 % | **B.E:** 228.23 and 231.5 eV  **R.I:**31.84 and 24.63 % | **-** | **B.E:** 160.80 and 161.78 eV  **R.I:**17.07 and 82.93 % |
| **Charged** | With additive:  **B.E:** 227.25 and 230.45 eV  **R.I:** 18.21 and 7.67 % | **B.E:** 228.54 and 231.62 eV    **R.I:**26.02 and 37.36 % | **B.E:** 161.77 and 162.95 eV    **R.I:**30.45 and 15.21 % | **B.E:** 160.81 and 161.99 eV  **R.I:**36.24 and 18.10 % |
|  | Without additive:  **B.E:** 227.13 and 230.33 eV  **R.I:** 66.75 and 3.76 % | **B.E:** 228.24 and 231.41 eV  **R.I:**11.97 and 13.70 % | **B.E:** 161.67 and 162.85 eV  **R.I:22.58** and 11.28 % | **B.E:** 160.70 and 161.89 eV  **R.I:28.41** and 14.19 % |

B.E=Binding energy, R.I=Relative peak intensity

**Table S1.** XPS Mo 3d_5/2-1/2_ and S 2p_3/2-1/2_ binding energies (eV) for charged and discharged cycled electrode.

**Figure S5.** *Ex-situ* XPS analysis of the Mo6S8 cathode: Mo3d spectra of (a) the fully discharged and (b)the fully charged cell, S 2s spectra of (c) the fully discharged and (d) the fully charged cell for electrolytes with additive (top) and without additive (bottom).

***Ex-situ* analysis of the anode**

To further elucidate the chemical composition of the interfacial layers formed on Mg anodes, XPS measurements were performed on electrodes harvested after 1 h of cycling, both with and without the TMSB additive. The Si 2p, Mg 2p, F 1s, and O 1s spectra are presented in **Figure S4.** The Si 2p spectra **(Figure S6a)** can be deconvoluted into two peaks at ~98 eV and ~102.95 eV, corresponding to elemental Si⁰ and oxidized SiOₓ species, respectively^[9-10]^ . The detection of Si-containing species only in the additive-containing electrolyte indicates that TMSB decomposition products are incorporated into the SEI as Si-based compounds. The Mg 2p spectra **(Figure S6b)** show contributions from Mg metal (~48.63 eV) and MgO/MgF_2_ (~50.52 eV). For the additive-free electrolyte, a stronger MgO/MgF_2_ signal is observed, consistent with the formation of a thick, passivating SEI. In contrast, the electrode cycled with TMSB exhibits a relatively higher Mg metal signal and reduced MgO/MgF_2_ content, suggesting the presence of a thinner and less passivating interfacial layer^[11]^. The F 1s spectra **(Figure S6c)** reveal peaks at ~685.5 eV (MgF_2_) and ~688.6 eV (−CFₓ), both of which originate from electrolyte decomposition and side reactions^[12]^. The reduced MgF_2_ signal in the additive-containing electrolyte indicates that TMSB suppresses parasitic fluorine-rich byproducts at the Mg surface. The O 1s spectra **(Figure S6d)** can be deconvoluted into peaks at ~530.3 eV (MgO), ~532 eV (Si−O/B−O), and ~533.2 eV (C−O)^[13]^. Compared with the additive-free cell, the TMSB-containing electrolyte shows a relative enhancement in Si−O features, corroborating the incorporation of siloxane/silanol fragments from TMSB degradation into the interfacial layer. Taken together, the XPS results demonstrate that TMSB fundamentally alters the Mg anode interphase chemistry. In the absence of TMSB, the anode surface is dominated by MgO and MgF_2_, which are electronically insulating and contribute to poor interfacial stability. By contrast, with TMSB, the SEI is thinner and enriched in Si-based species, while MgO/MgF_2_ formation is significantly suppressed. These findings are fully consistent with the Raman/TERS results, which revealed a siloxane-dominated interphase for the additive-containing system, in contrast to the thick, borate/oxide/carboxylate-rich SEI formed without TMSB. Thus, both spectroscopic techniques converge to show that TMSB promotes the formation of a stable, thin, and chemically distinct SEI that mitigates Mg passivation and supports long-term cycling stability.

**Figure S6.** *Ex-situ* XPS analysis of 1 hours cycled Mg anode: (a) XPS spectra of Si 2p for with additive (b) Mg 2p (c) F 1s and (d) O 1s regions for with additive (top XPS) and without additive (bottom XPS) cell.

1. **CV**

**Figure S7.** CV without additive: -1 V - 1 V (vs. Mg pseudo-reference), 2 mV/s , 10 cycles, starting from OCP towards the negative terminus. WE: Mg(0001), CE: glassy carbon RE: Mg-wire.


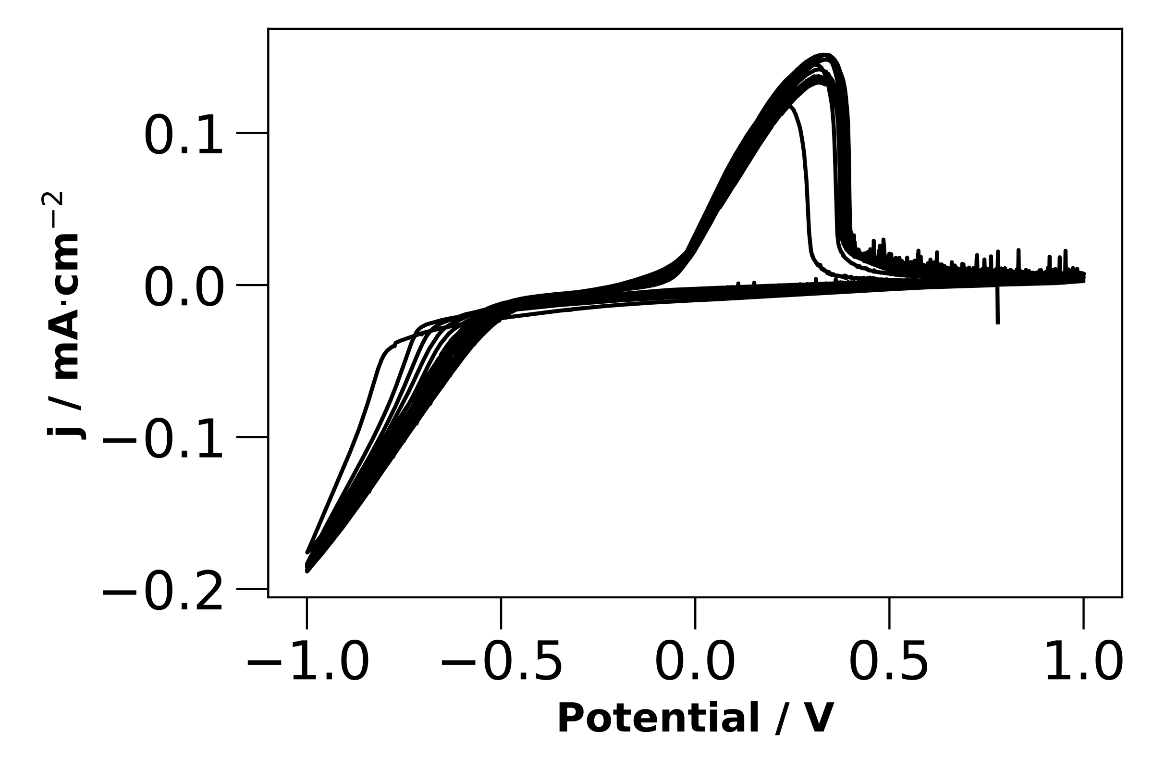


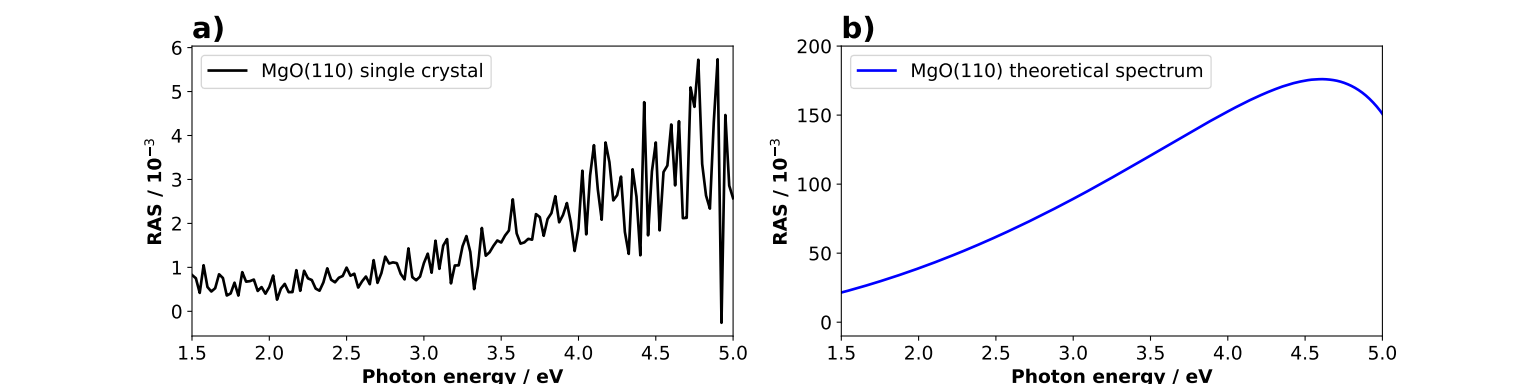


**Figure S8.** Reflection anisotropy spectra of a) an MgO(110) single crystal (Thermo Scientific) and b) a computational RA-spectrum of Mg(110). The latter was calculated via theYambo code using a 40 atom, geometry-optimised supercell, in analogy to the approach described elsewhere. Due to the non-ideality of the MgO(110) single crystal, the magnitude of the anisotropy is much lower and shows a higher signal-to-noise ratio than the computational spectrum, where an ideal single crystal (without defects) is used.

**Table S2.** Mg//Mo_6_S_8_ full cell performance from previous studies in comparison with our work.

| **Electrolyte additive** | **Electrolyte** | **C-rate/Current density** | **Capacity and cycle number** | **Reference** |
| --- | --- | --- | --- | --- |
| Zn(BH_4_)_2_ | 0.5 M Mg(TFSI)_2_/DME | 0.5 C | 40.8 mA h g^-1^ (100 cycles) | *ACS Energy Lett*. 2024,9,6, 2685-2695^[14]^. |
| H_3_PO_4_-SiCl_4_ | 0.5 M Mg(TFSI)_2_/DME | 0.1 C | ~ 80 mA h g^-1^ 40 cycles) | *Chem. Eng. J.* 2021, 426, 130751^[15]^. |
| EtBr_2_ | 0.5 M Mg(TFSI)_2_/G2 | 0.1 C | ~ 75 mA h g^-1^ (50 cycles ) | *ACS Nano* 2023, 17 (10), 8980-8991^[16]^. |
|  |  |  |  |  |
| CuCl_2_ | 0.5 M Mg(TFSI)_2_/G2 | 0.2 C | ~ 60 mA h g^-1^ (50 cycles) | *Chem. Eng. J.* 2022, 444, 136592^[17]^. |
| 1-methylimidazole | 0.3 M Mg(TFSI)_2_/DME | 0.2 C | ~ 50 mA h g^-1^ (250 cycles) | *ACS Appl. Mater. Interfaces* 2024, 16, 17673-17682^[18]^. |
| Tetrabutylammonium chloride (TBAC), | Mg(OTf)_2_/DME | 0.1 C | 59 mA h g^-1^ (200 cycles) | [*Energy Storage Materials*](https://www.sciencedirect.com/journal/energy-storage-materials) 2022,45, 1120-1132^[19]^. |
| Ga | 0.5 M Mg(TFSI)2/DME | 0.05 C | 90 mA h g-1 (10 cycles) | J. Mater. Chem. A, 2022,10,12104-12113^[20]^. |
| Mg(BH4)2 | 0.3 M Mg[B(hfip)4]2/DME | 50 mA g–1 | ~ 50 mA h g-1 (600 cycles) | ACS Appl. Mater. Interfaces 2021, 13, 28, 33123–33132^[21]^. |
| TMSB | 0.3 M Mg[B(hfip)_4_]_2_/DME | 1 C | 50 mA h g^-1^ (1000 cycles) | Our work |

**Table S3.** Mg//Mg symmetric cell performance from previous studies in comparison with our work.

| **Electrolyte additive** | **Electrolyte** | **Current** | **Overpotential and stability** | **Reference** |
| --- | --- | --- | --- | --- |
| CuCl_2_ | 0.5 M Mg(TFSI)_2_/G2 | 0.1 mA cm^-2^ | 450 mV (800 h) | Chem. Eng. J. 2022, 444, 136592^[17]^. |
| EtBr_2_ | 0.5 M Mg(TFSI)_2_/G2 | 1 mA cm^-2^ | 250 mV (250 h) | ACS Nano 2023, 17 (10), 8980-899^[16]^. |
| SnCl_4_ | 0.5 M Mg(TFSI)_2_/DME | 0.01 mA cm^-2^ | 250 mV (650 h) | National Science Review 2020, 7 (2), 333-34^[22]^. |
| SbCl_3_ | 0.5 M Mg(TFSI)_2_/DME | 1 mA cm^-2^ | 300 mV (350 h) | Adv. Funct. Mater. 2023, 33 (5), 2210639^[23]^. |
| SiCl_4_ | 0.5 M Mg(TFSI)_2_/DME | 0.1 mA cm^-2^ | 350 mV (600 h) | Energy Storage Mater. 2022, 46, 1-9^[24]^. |
| Ga | 0.5 M Mg(TFSI)_2_/DME | 0.1 mA cm^-2^ | 500 mV (500 h) | J. Mater. Chem. A 2022, 10 (22), 12104-12113^[20]^. |
| Bi(OTf)_3_ | 0.3 M Mg[B(hfip)_4_]_2_/DME | 1 mA cm^-2^ | > 300 mV (500 h) | ACS Appl. Mater. Interfaces 2021, 13, 31, 37044–37051^[12]^. |
| TMSB | 0.3 M Mg[B(hfip)_4_]_2_/DME | 0.1 mA cm^-2^ | 330 mV (1200 h) | Our work |

References

[1] D. M. Driscoll, N. K. Dandu, N. T. Hahn, T. J. Seguin, K. A. Persson, K. R. Zavadil, L. A. Curtiss, M. Balasubramanian, *Journal of The Electrochemical Society* **2020**, *167*, 160512.

[2] O. Holin, Y. Gofer, D. T. Major, D. Aurbach, *The Journal of Physical Chemistry C* **2023**, *127*, 14863-14873.

[3] N. Goutev, K. Ohno, H. Matsuura, *The Journal of Physical Chemistry A* **2000**, *104*, 9226-9232.

[4] S. Dinda, S. Trivedi, A. Roy, F. D. Pammer, M. Fichtner, *Advanced Energy Materials* **2023**, *13*, 2302176.

[5] Y. Gu, E.-M. You, J.-D. Lin, J.-H. Wang, S.-H. Luo, R.-Y. Zhou, C.-J. Zhang, J.-L. Yao, H.-Y. Li, G. Li, *Nature Communications* **2023**, *14*, 3536.

[6] K. I. Hadjiivanov, D. A. Panayotov, M. Y. Mihaylov, E. Z. Ivanova, K. K. Chakarova, S. M. Andonova, N. L. Drenchev, *Chemical Reviews* **2020**, *121*, 1286-1424.

[7] D. Long, *Journal of Raman Spectroscopy* **2004**, *35*, 905-905.

[8] M. Dekermenjian, A. P. Ruediger, A. Merlen, *RSC advances* **2023**, *13*, 26683-26689.

[9] X. Tai, X. Li, A. Kakimov, S. Li, W. Liu, J. Li, J. Xu, D. Li, X. Sun, *Journal of Materials Research* **2019**, *34*, 2425-2434.

[10] J. Zhang, D. S. Wavhal, E. R. Fisher, *Journal of Vacuum Science & Technology A: Vacuum, Surfaces, and Films* **2004**, *22*, 201-213.

[11] D. Chinnadurai, W. Y. Lieu, S. Kumar, G. Yang, Y. Li, Z. W. Seh, *Nano Letters* **2023**, *23*, 1564-1572.

[12] Z. Meng, Z. Li, L. Wang, T. Diemant, D. Bosubabu, Y. Tang, R. Berthelot, Z. Zhao-Karger, M. Fichtner, *ACS applied materials & interfaces* **2021**, *13*, 37044-37051.

[13] Z.-P. Zhuang, X. Dai, W.-D. Dong, L.-Q. Jiang, L. Wang, C.-F. Li, J.-X. Yang, L. Wu, Z.-Y. Hu, J. Liu, *Electrochimica Acta* **2021**, *393*, 139042.

[14] D. Zhang, Y. Sun, X. Liu, Y. Zhang, R. Wang, Y. Zhao, M. Pan, Y. Wang, S. Chen, M. Zhou, *ACS Energy Letters* **2024**, *9*, 2685-2695.

[15] R. Zhang, C. Cui, R. Li, Y. Li, C. Du, Y. Gao, H. Huo, Y. Ma, P. Zuo, G. Yin, *Chemical Engineering Journal* **2021**, *426*, 130751.

[16] A.-R. Jeon, S. Jeon, G. Lim, J. Jang, W. J. No, S. H. Oh, J. Hong, S.-H. Yu, M. Lee, *ACS nano* **2023**, *17*, 8980-8991.

[17] R. Zhang, C. Cui, R. Xiao, L. Ruinan, T. Mu, H. Huo, Y. Ma, G. Yin, P. Zuo, *Chemical Engineering Journal* **2022**, *444*, 136592.

[18] J. Xiao, X. Zhang, H. Fan, Q. Lin, Z. S. Ng, W. Chen, Y. Zhang, *ACS Applied Materials & Interfaces* **2024**, *16*, 17673-17682.

[19] D.-T. Nguyen, A. Y. S. Eng, R. Horia, Z. Sofer, A. D. Handoko, M.-F. Ng, Z. W. Seh, *Energy Storage Materials* **2022**, *45*, 1120-1132.

[20] C. Pechberty, A. Hagopian, J.-B. Ledeuil, D. Foix, J. Allouche, J.-N. Chotard, O. Lužanin, J. Bitenc, R. Dominko, R. Dedryvère, *Journal of Materials Chemistry A* **2022**, *10*, 12104-12113.

[21] Z. Li, T. Diemant, Z. Meng, Y. Xiu, A. Reupert, L. Wang, M. Fichtner, Z. Zhao-Karger, *ACS applied materials & interfaces* **2021**, *13*, 33123-33132.

[22] R. Lv, X. Guan, J. Zhang, Y. Xia, J. Luo, *National science review* **2020**, *7*, 333-341.

[23] Y. Li, G. Yang, C. Zhang, W. Y. Lieu, C. Y. J. Lim, S. Sun, J. Wang, S. Jiang, Z. Xing, Z. Sofer, *Advanced Functional Materials* **2023**, *33*, 2210639.

[24] Y. Li, X. Zhou, J. Hu, Y. Zheng, M. Huang, K. Guo, C. Li, *Energy Storage Materials* **2022**, *46*, 1-9.
